# Exploring CYP17 Gene Polymorphism as a Predictive Marker in Iraqi Women with Polycystic Ovary Syndrome and Its Association with Hormonal Dysregulation: CYP17A1 Polymorphism in Iraqi women with PCOS

**DOI:** 10.31661/gmj.vi.3746

**Published:** 2025-06-03

**Authors:** Saleh Ali Alqadoori, Nour Saeed Hassan, Farah Ali Dawood

**Affiliations:** ^1^ Department of Medical Laboratory Techniques, Balad Technical Institute, Middle Technical University, Baghdad, Iraq; ^2^ Preparatory of OM-ALTobool Vocational, Department of Second Vocational Education at Al-karkh, The General Direction for Vocational Education, Baghdad, Iraq; ^3^ Department of Basic Science, College of Dentistry, Mustansiriah University, Baghdad, Iraq

**Keywords:** Polycystic Ovary Syndrome, CYP17 Gene, Polymorphism, Anti-Müllerian Hormone

## Abstract

**Background:**

Although most women of reproductive age diagnosed with polycystic ovary syndrome (PCOS) represent a common disorder with significant long-term health implications. Investigations on the CYP17A1 gene, which plays a pivotal role in androgen biogenesis, explored its potential role as a predictive marker for the risk of PCOS. This research was conducted to evaluate the association of CYP17A1 polymorphism (rs743572 variant) with susceptibility to polycystic ovarian syndrome (PCOS) among the Iraqi population.

**Materials and Methods:**

We executed a case-control study consisting of 66 PCOS patients and 74 controls. Restriction Fragment Length Polymorphism (RFLP) was used to detect the genotypes.

**Results:**

The TT genotype of CYP17A1 was significantly associated with an increased risk of PCOS, with an approximately fourfold higher odds of developing PCOS versus the CC genotype. No significant increase in risk was seen for the CT genotype. The TT polymorphism of CYP17A1 (rs743572) was significantly associated with PCOS (adjusted OR=3.97, P=0.03), while the CT variant showed a non-significant trend, after adjusting for age and BMI.

**Conclusion:**

The current study, by providing further evidence for the association between CYP17A1 genetic polymorphisms and PCOS in multi-ethnic populations, has important implications for the management of this complex disorder, indicating the potential use of genotyping in assessing the genetic risk of the disorder in different ethnic groups.

## Introduction

Polycystic ovary syndrome (PCOS) is a prevalent reproductive endocrine disorder, affecting about five to ten percent of women of reproductive age (Goodarzi et al., 2011). PCOS is a clinical diagnosis based on parameters including obesity, oligomenorrhea or amenorrhea, elevated androgen levels, and impaired ovulation (Akhtar et al., 2005; Apridonidze et al., 2005). Additionally, women with PCOS exhibit reduced aromatase activity, and due to the relative decrease in follicle-stimulating hormone (FSH) output, follicular growth is disrupted, leading to excess androgen accumulation and hyperandrogenism (Nelson et al., 1999). Defects in the steroidogenic pathway and cortisol metabolism cause excessive adrenal androgen production, which affects about thirty percent of women (Goodarzi et al., 2015). Thus, hyperandrogenism appears central to the pathophysiology of PCOS, influencing both its metabolic and reproductive components (Chaudhary et al., 2021), given that theca cells are the primary source of androgens in PCOS (Jakubowicz et al., 1997; Nestler et al., 1998).

In theca cells, excessive androgen biosynthesis in PCOS is linked to upregulated expression of the CYP17A1 gene, specifically steroid-17-α-hydroxylase/17,20-lyase (Wickenheisser et al., 2012). CYP17, a member of the cytochrome P450 family 17, plays a critical role in androgen synthesis and encodes enzyme activities such as 17-α-hydroxylase and 17,20-lyase (Jahromi et al., 2016). CYP17 is expressed in thecal cells, adrenal glands, and Leydig cells (Chua et al., 2011).

A single nucleotide polymorphism (SNP) in the CYP17 gene involves the substitution of thymine (T) with cytosine (C) at 34 bp upstream of the transcription start site in the promoter region (Carey et al., 1994). This may create an additional Sp-1 transcription factor binding site, potentially increasing CYP17 expression (Carey et al., 1993) due to functional changes in SNP-mediated gene regulation (Xing et al., 2022).

Both adrenal glands and ovaries produce androgens, the final products of enzymatic reactions converting cholesterol to dehydroepiandrosterone (DHEA) and androstenedione. Rate-limiting enzymes in these tissues regulate sex steroid synthesis (Wawrzkiewicz et al., 2020). Variations in the activity of these enzymes contribute to phenotypic diversity in androgen levels (de Medeiros et al., 2015). Androgen biosynthesis may also be influenced by mutations in steroidogenic pathway genes, such as CYP1A, CYP3, CYP11, CYP17, CYP19, and CYP21 (de Medeiros et al., 2015; Pigny et al., 2019).

Previous genetic association studies suggest that hereditary factors significantly influence PCOS susceptibility, with multiple genetic variants linked to the disorder (Saddick et al., 2020). Candidate gene studies provide insights into differences between patient and control populations (Douma et al., 2019). However, only a few studies have examined the association between CYP17 polymorphisms and PCOS risk (Chen et al., 2010; Cong et al., 2018; Li et al., 2015).

Anti-Müllerian hormone (AMH), produced by granulosa cells of small ovarian follicles, regulates folliculogenesis (Carlsson et al., 2006; Dumont et al., 2015) and inhibits CYP17, modulating steroidogenesis (Teixeira et al., 1999). Conversely, some studies suggest that elevated AMH levels contribute to ovarian dysfunction (Laven et al., 2004). In PCOS, abnormally high AMH impairs FSH-stimulated follicular development, leading to anovulation (Pigny et al., 2007).

Another hallmark of PCOS is increased GnRH pulsatility, causing hypersecretion of luteinizing hormone (LH), particularly in lean women with oligomenorrhea (Pellatt et al., 2011). A high LH/FSH ratio (often 3:1) is common, with LH levels two- to three-fold higher than FSH. This elevated ratio enhances LH responsiveness to GnRH (Hendriks et al., 2008).

Few studies have explored the relationship between PCOS and CYP17A1 polymorphisms (Munawar Lone et al., 2021; Ashraf et al., 2020), and data from Middle Eastern populations, remain scarce, particularly Iraqi women.

As Middle Eastern populations exhibit unique genetic admixtures and may modulate have different penetrance of CYP17A1 variants compared to Europeans (Bao); Despite the well-documented role of CYP17A1, 3β-HSD, and CYP11A in androgen biosynthesis and their association with PCOS, the ethnic-specific genetic landscape of PCOS, particularly in understudied populations like Iraqi women, remains poorly characterized. By integrating genotype-phenotype correlations in a homogeneous ethnic group, our work not only validates the universality of CYP17A1’s role in PCOS but also tries to seek for population-specific risk patterns.

## Materials and Methods

**Figure-1:**
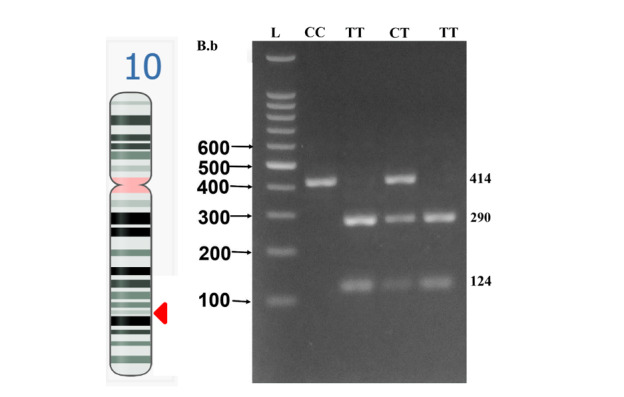
Figure 1. NCBI Genome Data Viewer screenshot of CYP17A1 gene location on chromosome 10 (left side); Detection of CYP17 gene (rs743572) after digestion with MspA1 (right side). The MspA1 enzyme produced three bands of 414 bp (continuous band), 290 bp, and 124 bp in the case of the CT genotype when it digested the CYP17 gene amplicon (414 bp). While samples with homozygous CC produced just one band of 414 bp, samples with the TT genotype produced two bands of 290 bp and 124 bp

### Study Design

This was a case-control study aimed at investigating the association between CYP17A1 gene polymorphism and the risk of polycystic ovary syndrome (PCOS) in Iraqi women. The study was conducted from June 2023 to October 2024 at Baghdad Teaching Hospital, Iraq.

### Participants

A total of 140 women (66 PCOS patients and 74 healthy controls), aged 14-49 years, were included. PCOS diagnosis followed the Rotterdam criteria (2003).

Exclusion criteria for PCOS patients included hyperandrogenism due to other conditions, such as 21-hydroxylase deficiency, non-classical adrenal hyperplasia, hyperprolactinemia, or androgen-secreting tumors. Healthy controls were selected from the same hospital’s gynecology department, with no history of PCOS, infertility, or menstrual irregularities.

### Clinical and Hormonal Assessment

Clinical and demographic data were collected through physical examinations and structured interviews. Hormonal profiles (LH, FSH, and AMH) were assessed using fasting blood samples collected on days 3-5 of the menstrual cycle (or at any time for participants with amenorrhea), after a 12-hour fast.

Luteinizing hormone (LH) and follicle-stimulating hormone (FSH) levels were quantified using the Maglumi chemiluminescent immunoassay (CLIA) kit, with confirmatory measurements performed on the Beckman Coulter Immunoassay System Access 2.

To ensure the reliability and reproducibility of our assays, both intra-assay and inter-assay coefficients of variation were maintained below 10%.

### Genetic Analysis

Genomic DNA was isolated from 5 mL of peripheral blood samples using the ReliaPrep™ Blood DNA Miniprep System (A5081). The CYP17A1 rs743572 polymorphism was genotyped using the established PCR-RFLP method (Azziz, 2006).

This method was selected for its enhanced sensitivity in detecting genetic variants, a critical factor in genetic association studies.

### PCR Reaction Components

The 25 μL PCR reaction mixture contained: 50 ng of genomic DNA extracted from participant blood samples; 10 μM each of forward (5ʹ-CATTCGCACTCTGGAGTC-3ʹ) and reverse (5ʹ-AGGCTCTTGGGGTACTTG-3ʹ) primers; Nuclease-free water to adjust the final volume; 2× GoTaq® Green Master Mix (M712, Promega, USA), which includes Taq DNA polymerase, dNTPs, and reaction buffer.

The circumstances of the thermal cycling for PCR amplification were as follows: Initial denaturation at 95°C for 3 min to ensure that the DNA template was fully denatured.

This was followed by 30 cycles of amplification, each consisting of: DNA strand separation through denaturation at 94°C for 46 s, 46 s of annealing at 55°C, and a one-minute extension at 72°C. Afterward, the PCR results were loaded into a 2% agarose gel and incubated at 72°C for 5 additional minutes to complete the extension step.

Next, the results were mixed with the restriction enzyme MspA1. The digested products were subsequently separated using a 2% agarose gel, and finally, the two profiles were visualized under ultraviolet light following staining with a suitable DNA dye.

Genotypes were defined according to the sizes of the fragments detected: The TT genotype was detected as two bands, one of 290 base pairs (bp) and the other of 124 bp. In the CC genotype, only one band was observed at 414 bp, since it lacked the restriction enzyme recognition site. Genotypes CT and TT generated three bands (414 bp, 290 bp, 124 bp), indicating heterozygosity.

Using this approach, the genotypic distributions of the CYP17A1 polymorphism (rs743572) in the study population could be accurately assessed, allowing for evaluation of any potential association with the risk of developing PCOS among the Iraqi women included in this research.

Figure-1 shows the CYP17A1 gene location on chromosome 10. Data on the samples were collected until October 2023, after which the genetic predisposition was determined, and the significance of associations between the gene polymorphism and the clinical outcomes of the studied patients were evaluated.

### Statistical Analysis

The data analysis in this study was conducted using IBM SPSS Statistics version 22 (Armonk, New York, USA) and GraphPad Prism 10. Independent t-tests and Mann-Whitney U tests were used for comparing continuous variables, with the choice of test depending on the data distribution. Logistic regression models, both adjusted and unadjusted, were utilized to determine odds ratios (OR) and 95% confidence intervals (CI) for the association between CYP17A1 genotypes and the risk of Polycystic Ovary Syndrome (PCOS). Furthermore, the genotypic distributions in both groups were assessed for deviation from Hardy-Weinberg equilibrium to evaluate the validity of the genetic analysis.

### Ethical Approval and Consent

All participants provided written informed consent before being enrolled in the study. Ethical approval was obtained from college of Health and Medical Technology /University of Al-Turath (CSEC/1019/0004).

## Results

**Table:** Table1. Comparison of Clinical and Hormonal Variables between PCOS Patients and Healthy Controls.

**Variable**	**Patient** **Mean ± SD**	**Control** **Mean ± SD**	**T-Test P-Value**	**MannWhitney U** **P-Value**
**Age (years)**	27.59 ± (3.54)	26.77 ± (3.01)	0.15	0.13
**BMI** **(kg/m^2^) **	25.38 ± (3.07)	23.55 ± (1.38)	0.01^*^	0.01^*^
**LH**	12.61 ± (2.71)	6.80 ± (3.25)	0.01^*^	0.01^*^
**FSH**	5.88 ± (3.23)	7.57 ± (1.87)	0.01^*^	0.01^*^
**LH/ FSH**	2.71± (1.3)	0.97 ± 0.57	0.01^*^	0.01^*^
**AMH**	9.24 ± (1.97)	4.03 ± (0.94)	0.01^*^	0.01^*^

**BMI:** body mass index; **FSH:** follicle stimulating hormone; **LH:** luteinizing hormone. LH/ FSH ratio, **AMH:** Anti-Müllerian hormone. ^*^The logistic regression test was applied, and “statistical significance was set at P-value<0.05”

**Table:** Table2. Determination of CYP17A1 (rs743572) Association with PCOS Patients and Controls using Different Genetic Models

**CYP17A1 (rs743572)**	**Control**	**PCOS**	**Crude Odds Ratio**	**Adjusted #Odds Ratio**	**Adjusted Lower 95% CI**	**Adjusted Upper 95% CI**	**Crude P-value**	**Adjusted P-value**
**CC**	46 (62.16%)	29 (43.94%)	1.0	1.0	-	-	-	-
**CT**	22 (29.73%)	24 (36.36%)	1.73	1.87	0.84	4.19	0.19	0.13
**TT**	6 (8.11%)	13 (19.70%)	3.44	3.97	1.18	13.31	0.04*	0.03*

# adjusted based on the age and BMI. ^*^The logistic regression test was applied, and statistical significance was set at P-value <0.05

### Phenotypic and Anthropometric Features

Significant differences (P<0.01) were observed in multiple parameters between the PCOS and control groups, including body mass index (BMI, kg/m²), anti-Müllerian hormone (AMH), luteinizing hormone (LH), follicle-stimulating hormone (FSH), and the LH/FSH ratio. Detailed results are presented in Table-1.

The analysis revealed statistically significant differences between patients and controls for several variables. Both the t-test and Mann-Whitney U test showed no significant age difference between groups (P=0.155 and P=0.129, respectively), with patients having a mean age of 27.59 years (SD=3.54) compared to 26.77 years (SD=3.01) in controls. However, BMI differed significantly (t-test: P=0.000021; Mann-Whitney U test: P=0.000147), with patients exhibiting a higher mean BMI (25.38 ± 3.07) than controls (23.55 ± 1.38).

LH levels were significantly elevated in patients compared to controls (t-test: P<0.0001; Mann-Whitney U test: P<0.0001), with mean values of 12.61 (SD=2.71) versus 6.80 (SD=3.25), respectively. F

SH levels also differed significantly (t-test: P=0.000346; Mann-Whitney U test: P=0.000017), with patients showing lower mean FSH levels (5.88 ± 3.23) compared to controls (7.57±1.87).

The LH/FSH ratio was significantly higher in patients (t-test: P<0.0001; Mann-Whitney U test: P<0.0001), with a mean of 2.71 (SD=1.3) versus 0.97 (SD=0.57) in controls. AMH levels were also markedly elevated in patients (9.24±1.97) compared to controls (4.03±0.94), with both tests confirming high significance (P<0.0001).

Further statistical analysis confirmed significant differences between patients and controls for key variables. Age did not differ significantly (t-test: P=0.15; Mann-Whitney U test: P=0.13), with mean ages of 27.59 (SD=3.54) and 26.77 (SD=3.01) years, respectively. However, BMI was significantly higher in patients (25.38±3.07 vs. 23.55±1.38; t-test: P=0.000021; Mann-Whitney U test: P=0.000147).

LH levels were substantially higher in patients (12.61±2.71 vs. 6.80±3.25; both tests: P<0.0001), whereas FSH levels were lower (5.88±3.23 vs. 7.57±1.87; t-test: P=0.000346; Mann-Whitney U test: P=0.000017). AMH levels were significantly elevated in patients (9.24±1.97 vs. 4.03±0.94; both tests: P<0.0001).

### Genotypic Frequency Distribution

The analysis revealed differences in the distribution of gene polymorphism frequencies between patient and control groups, using the CC polymorphism as the reference. The CC polymorphism was found in 43.9% of patients (29/66) and 62.2% of controls (46/74). This polymorphism served as the baseline reference (OR=1.00), as shown in Table-2.

For the TT vs. CC comparison, the TT polymorphism was present in 19.7% of patients (13/66) and 8.1% of controls (6/74). The unadjusted (crude) OR for TT was 3.44 (95% CI: 1.18-13.31, P=0.04), indicating that patients were 3.44 times more likely to carry the TT polymorphism than controls, a statistically significant association. After adjusting for confounding factors (age and BMI), the adjusted OR increased to 3.97 time (P=0.03), further supporting a significant independent association between the TT polymorphism and patient status.

For the CT vs. CC comparison, the CT polymorphism was observed in 36.4% of patients (24/66) and 29.7% of controls (22/74). The crude OR for CT was 1.73 (95% CI: 0.84-4.19, P=0.19), suggesting a higher but non-significant prevalence of CT in patients compared to controls. After adjustment, the OR remained non-significant (adjusted OR=1.87, P=0.113), reinforcing that while CT polymorphism may be more frequent in patients, the association lacks statistical significance after accounting for age and BMI.

## Discussion

PCOS is among the most widespread endocrine disorders in females. Obesity is one of the key predisposing risk factors contributing to PCOS development (Rahimi & Mohammadi, 2019). Our study found a highly significant difference in BMI between patients and controls, supporting this association. A study by Legro (2012) reported that obesity correlates with hypothalamic-pituitary-ovarian dysfunction, promoting PCOS development. Increased obesity leads to elevated androgen production, further stimulating luteinizing hormone (LH) and contributing to hyperandrogenism (Güngör et al., 2023).

Our findings align with previous studies (Glueck & Goldenberg, 2019; Güngör et al., 2023; Al-Lami et al., 2020), which also reported significant BMI differences between PCOS patients and controls. Regarding LH levels, our study observed a highly significant increase in PCOS patients compared to controls, consistent with other research (Güngör et al., 2023; Al-Lami et al., 2020; Munawar et al., 2021). While most PCOS patients exhibit elevated LH levels, some show no significant changes in LH, FSH, or the LH/FSH ratio yet still meet diagnostic criteria. Typically, women with PCOS have higher serum LH concentrations (Ashraf et al., 2020), attributed to episodic LH secretion and increased pulse frequency.

Additionally, elevated LH may result from heightened gonadotropin-releasing hormone (GnRH) secretion or pituitary hypersensitivity to GnRH due to abnormal ovarian feedback (Malini & George, 2018).Our study also revealed a highly significant elevation in anti-Müllerian hormone (AMH) levels in PCOS patients, consistent with prior research (Güngör et al., 2023; Hassan, 2010; Jabr & Al-Hakeim, 2015). The rise in AMH levels in PCOS is linked to increased AMH production per follicular unit and the presence of small antral follicles, making it a potential diagnostic marker for PCOS or polycystic ovarian morphology (PCOM) (Liu et al., 2019).

We assessed the association between different CYP17A1 genotypes (CT and TT) and PCOS risk, using the CC genotype as the reference. Both crude (unadjusted) and adjusted (for age and BMI) odds ratios were analyzed. The TT genotype showed a significant association with PCOS in both analyses, suggesting that individuals with this genotype may have a higher PCOS risk than those with the CC genotype.

This association implies that the TT polymorphism may upregulate CYP17A1 gene expression, leading to excessive androgen production—a hallmark of PCOS-related hyperandrogenism. These findings support previous studies identifying elevated androgen levels as a key factor in PCOS pathogenesis (Goodarzi et al., 2015). The rs743572 polymorphism, located in the CYP17A1 promoter region, introduces an additional Sp-1 transcription factor binding site when T replaces C (Carey et al., 1994).

This may result in enhanced transcription of the CYP17A1 gene, leading to excessive androgen production, a key feature of PCOS. The TT genotype showed the strongest association with PCOS risk, supporting our hypothesis. A meta-analysis of 10 studies (CT vs CC genotypes) reported an odds ratio=2.25 (CI=1.338-3.778; P=0.002), indicating the CT genotype had 2.25-fold higher risk than CC. These findings agree with studies in other populations (Liu et al., 2021; Ashraf et al., 2020).

Our study found a significant association between the TT genotype of rs743572 and PCOS risk, consistent with (Hoyos et al., 2020). Unlike some Asian population studies that reported stronger association with the CT genotype (Li et al., 2015), we found no statistically significant PCOS risk with the CT genotype. This suggests CYP17A1 rs743572 genotyping could help identify high-risk women, particularly TT genotype carriers. Early identification may enable personalized management strategies, including lifestyle interventions or anti-androgen therapies.

Another meta-analysis confirmed the TT genotype's significant association with higher PCOS risk, particularly under dominant and codominant models, supporting our findings (Albedairy et al., 2024).

Studies on Iraqi women also reported higher TT genotype frequency in PCOS patients (Hoyos et al., 2020). Furthermore, genetic studies in North Indian and Iranian populations demonstrated strong links between TT genotype and PCOS, further validating our results (Liu et al., 2021).

BMI is a known risk factor for PCOS. Here, we controlled for BMI to isolate the effect of genotypes on PCOS risk. The adjusted odds ratio for BMI (1.38) confirms it as an independent risk factor, with each unit increase in BMI associated with 1.38-fold higher PCOS risk. After adjusting for BMI and age, the TT genotype remained significantly associated with PCOS, indicating its risk effect is independent of these factors. This further supports the role of genetic susceptibility in PCOS pathogenesis.

While BMI confounds the relationship between genotype and PCOS risk, our analysis accounts for BMI, allowing us to assess the direct association between genotypes and PCOS. Our findings align with existing literature demonstrating strong links between elevated BMI and PCOS prevalence. Notably, studies consistently show that higher BMI exacerbates PCOS-related reproductive and metabolic dysfunction. For instance, cross-sectional studies report that obesity worsens both reproductive abnormalities (e.g., menstrual dysfunction, hyperandrogenism) and metabolic complications (e.g., insulin resistance, dyslipidemia) (Heidarzadehpilehrood et al., 2022; Gill et al., 2023).

Genetic evidence further supports our results. Genome-wide association studies (GWAS) reveal that PCOS-associated genetic variants have stronger effects in individuals with higher BMI (Ma et al., 2021). This highlights the critical interplay between genetic predisposition and obesity in driving PCOS development and severity, consistent with our conclusions.

Age is also considered a risk factor for PCOS. In our analysis, the adjusted odds ratio for age (1.05, P=0.427) suggests a minimal, non-significant contribution to PCOS risk after accounting for genotype and BMI. While age itself shows limited independent impact on PCOS risk in this dataset, adjusting for it helps isolate genotype-specific effects.

The crude (unadjusted) odds ratios reflect the genotype-PCOS relationship without accounting for BMI or age. This unadjusted approach does not control for BMI’s influence on PCOS risk, meaning observed associations may partly reflect BMI differences across genotypes. In contrast, the adjusted analysis isolates the genotype effect by controlling for BMI and age.

For example, the adjusted odds ratio of 2.2 (95% CI: 1.5-3.1) for overweight vs. normal weight individuals (Table-2) indicates that both genotype and BMI contribute to PCOS risk in the studied population.

These methodological considerations align with existing literature. For instance, a study used Mendelian randomization to assess BMI’s causal role in PCOS, emphasizing that BMI-adjusted analyses are essential for accurate genetic risk estimation (Burns et al., 2024).

Similarly, a study on PCOS-endometrial cancer links highlighted how multivariate adjustment (e.g., for age/BMI) clarifies associations amidst statistical uncertainty (Zhao et al., 2020). Such approaches ensure robust genetic risk estimates, reinforcing that adjusted odds ratios and confidence intervals are critical in PCOS research (Lu et al., 2023; Boldis et al., 2024).

## Conclusion

This study reveals variations in key anthropometric and hormonal parameters between PCOS patients and controls. Patients with PCOS exhibited higher BMI, higher LH, reduced FSH, and markedly increased AMH levels compared to controls.

Genetic analysis revealed a significant association between the TT genotype and increased PCOS risk, with nearly fourfold higher odds versus the CC genotype. Conversely, the CT genotype showed a non-significant trend toward higher risk, which disappeared after adjustment. These results contribute to growing evidence on PCOS genetics and highlight CYP17A1's potential as a predictive marker.

## Conflict of Interest

The authors declare no conflict of interests.
